# Microalgal and Cyanobacterial Biomasses Modified the Activity of Extracellular Products from *Bacillus pumilus*: An In Vitro and In Vivo Assessment

**DOI:** 10.1007/s12602-024-10350-z

**Published:** 2024-09-11

**Authors:** Jorge García-Márquez, Alba Galafat Díaz, Luis Molina-Roque, Marta Domínguez-Maqueda, Verónica de las Heras, Paula Simó-Mirabet, Antonio J. Vizcaíno, Juan Antonio Martos-Sitcha, Francisco Javier Alarcón-López, Miguel Ángel Moriñigo, María Carmen Balebona

**Affiliations:** 1 Departamento de Microbiología, Facultad de Ciencias, Instituto Andaluz de Biotecnología y Desarrollo Azul (IBYDA), Universidad de Málaga, CEI⋅MAR-Universidad de Málaga, 29071 Málaga, Spain; 2 Departamento de Biología y Geología, Universidad de Almería, CEI⋅MAR-Universidad de Almería, 04120 Almería, Spain; 3 Departamento de Biología, Facultad de Ciencias del Mar y Ambientales, Instituto Universitario de Investigación Marina (INMAR), Universidad de Cádiz, CEI⋅MAR-Universidad de Cádiz, 11510 Cádiz, Spain; 4 Grupo de Investigación en Acuicultura (GIA), IU-ECOAQUA, Universidad de Las Palmas de Gran Canaria, 35214 Telde, Las Palmas de Gran Canaria, Islas Canarias Spain; 5 LifeBioencapsulation S.L. El Alquián, 04131 Almería, Spain

**Keywords:** 3R principle, Aquaculture, Extracellular products, Microalgae, *Sparus aurata*

## Abstract

This study investigates the postbiotic potential of extracellular products (ECPs) from *Bacillus pumilus* strains cultivated on microalgae-supplemented media. We assessed enzymatic and antimicrobial activities to select ECPs that enhance the digestive processes in gilthead seabream. Additionally, we explored the in vitro enzymatic capacity of the chosen postbiotics to hydrolyze macromolecules in microalgae. Finally, a feeding trial was conducted to determine the in vivo effects of the ECPs on *Sparus aurata*. In vitro enzymatic assays demonstrated diverse hydrolytic capacities among ECPs. All conditions exhibited antimicrobial activity against *Photobacterium damselae* subsp. *piscicida*, with variation in inhibitory effects against *Vibrio harveyi* and *Tenacibaculum maritimum*. Furthermore, in vitro assays revealed differences in protein hydrolysis and soluble protein concentration, influencing amino acid and reducing sugar release from microalgal biomass. These analyses facilitated a selection to test ECPs in vivo. Lastly, the in vivo experiment revealed no differences in the growth performance, nutrient utilization, and general metabolism of *S. aurata* fed the experimental diets. Dietary inclusion of postbiotics increased the activity of key digestive enzymes in fish compared to the control group, and particularly, values increased significantly when the fish were fed with the ECP-nanoparticulate-supplemented diet. In conclusion, the inclusion of microalgae in the culture media significantly influences the activity of extracellular products from *B. pumilus* strains, as evidenced in both in vitro and in vivo assays.

**Supplementary Information:**

The online version contains supplementary material available at 10.1007/s12602-024-10350-z.

## Introduction

Aquaculture is a rapidly growing sector in feed production [1]. However, the reliance on fishmeal and fish oil as key feed ingredients poses a limitation to its expansion [2]. Microalgae have gained significant attention as a potential alternative ingredient in aquafeed due to their nutritional composition [3]. They are rich in proteins, lipids, and n-3 long-chain polyunsaturated fatty acids [4], providing a well-balanced amino acid profile [5]. Nonetheless, certain microalgae species possess recalcitrant cell walls that hinder the accessibility of intracellular nutrients, thereby limiting their use in aquafeeds [6, 7]. Thus, the fish’s ability to hydrolyze the microalgae cell wall will depend on its chemical composition and the fish’s digestive enzymatic activity [8].

Recently, a clear focus has been pointed out on the development of health products that utilize postbiotics, where emerging evidence suggests that bacterial viability may not be essential for beneficial effects on the host [9]. Postbiotics are non-viable bacterial products or metabolic by-products, including bacteriocins, organic acids, extracellular products, or enzymes, among others, that exhibit beneficial biological activity on the host [10]. Understanding of postbiotics and their potential health benefits has been growing in recent years [11]. Postbiotics can contribute to host health by improving specific physiological functions, although the exact mechanisms have not been fully clarified. For instance, postbiotics have shown promising properties in terms of hydrolytic and antagonistic capabilities, leading to biological responses that prevent intestinal diseases and microbial illnesses in farmed fish [12]. Additionally, postbiotics have demonstrated the ability to improve growth performance, modulate gut microbiota composition and function, and mitigate dysbiosis in aquaculture [13, 14]. However, the production of postbiotics still poses challenges due to limited knowledge of preparation and analysis methods, as well as the factors influencing their production [15].

The production and properties of postbiotics are largely influenced by factors such as bacterial strains, culture medium, bacterial treatment, and growth phase [11, 16]. For example, modifying the composition of the culture medium has been found to enhance the bacteriocin-inhibitory activity of postbiotics, as observed with the addition of glucose and yeast extract to a modified De Man-Rogosa-Sharpe (MRS) medium used for *Lactobacillus plantarum* I-UL4 [17]. Dairy-derived ingredients, like low-heat milk and milk permeate, have also been optimized as fermentation media for *Lactobacillus* spp. to produce postbiotic antifungal solutions [15]. In addition, exploring different culture media for postbiotic production could modulate their bioactivity, creating new opportunities for applications in biotechnology, particularly within the aquaculture and aquafeed sectors [11, 16]. In this context, microalgae have emerged as a highly promising substitute for conventional carbon sources in bacterial growth media, providing a wide range of nutrients to bacterial metabolism that can enhance or maximize the production of postbiotics with diverse activities [18, 19].

In recent years, several studies have been conducted on the in vitro hydrolysis of microalgae protein by the fish digestive system [7, 8, 20]. In vitro digestibility methods provide a quick, relatively simple, and cost-effective alternative to in vivo trials [21]. Moreover, these methods align with ethical considerations and the 3R principle (Directive 2010/63/UE) as they eliminate the need for animal use in experimentation. Thus, in vitro’s digestive simulations can provide valuable insights into the enzymatic capacity of postbiotics to hydrolyze macromolecules in microalgae and aid in the selection of postbiotics with higher proteolytic activity for further in vivo trials. However, to our knowledge, no research has assessed the in vitro microalgae hydrolysis by postbiotics.

In a previous study, our research group characterized several bacterial isolates from the gilthead seabream (*Sparus aurata*) intestinal tract fed a microalgae blend and proposed bacterial isolates UMA169 and UMA216 as putative probiotics [22]. The two isolates stood out for their extracellular enzyme activity and antagonism against several fish pathogens and were identified as different strains of *Bacillus pumilus*. In this piece of research, we investigated the postbiotic potential of extracellular products (ECPs) obtained from these two candidate probiotics grown on different microalgae-supplemented mediums. We evaluated their enzymatic and antimicrobial activity and aimed to select diverse ECPs with activities that enhance the digestive process of gilthead seabream. Additionally, we assessed the in vitro enzymatic capacity of the selected postbiotics to hydrolyze macromolecules in microalgae. Finally, a 2-month feeding trial was conducted to determine the in vivo effects of the ECPs on the growth performance, metabolic response, and intestinal enzyme activity in *S. aurata* juveniles.

## Material and Methods

### Bacterial Strain and Culture Conditions

*Bacillus pumilus* UMA169 and UMA216 were isolated from the gastrointestinal tract of gilthead seabream specimens fed with a diet containing a blend of microalgae and used in this research due to their in vitro enzymatic and antimicrobial activities [22]. The strains were cultured on tryptic soy agar (TSA, Oxoid Ltd., Basingstoke, UK) supplemented with 1.5% NaCl at 23 °C for 24 h. Then, one to two colonies were cultured on 50 mL of tryptic soy broth (TSB, Oxoid Ltd.) supplemented with 1.5% NaCl at 23 °C for 36 h (10^9^ colony-forming units (cfu) mL^−1^, start of the stationary phase) on shaking at 80 rpm.

### Microalgae Production

Microalgal biomasses (*Chlorella vulgaris*, *Microchloropsis gaditana*, *Tisochrysis galbana*, and *Arthrospira platensis*) were provided by LifeBioencapsulation S.L. (Spin-off; Universidad de Almería, Almería, Spain). After cultivation, harvesting, freeze-drying, and milling, the resulting powder was stored at − 20 °C until used.

### Extracellular Product Extraction

**Fig. 1:**
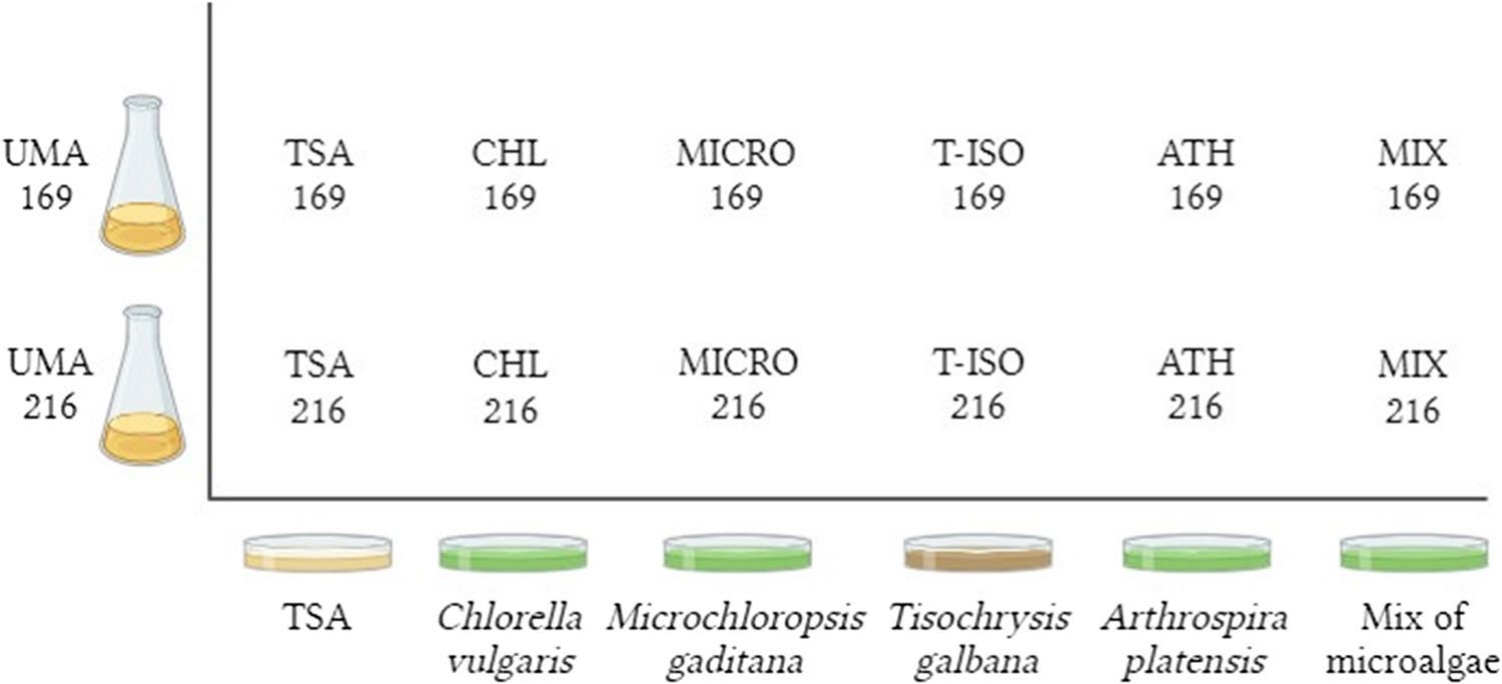
Different conditions for ECP extraction and nomenclature used in this experiment. Internal controls are not shown in the figure but they were named as their respective conditions but adding “Control” to them (e.g., Control TSA 169, Control CHL 169)

Extracellular products (ECPs) from a solid medium were obtained using the cellophane plate technique described by Liu [23]. Briefly, 1 mL of each suspension of bacterial strain described above was spread over sterilized cellophane sheets placed on TSA plates with 1.5% NaCl that was used as a control medium. Additionally, 1 mL was spread on solid medium plates (1.5% agar) supplemented with the following: (i) 5% *C. vulgaris*, (ii) 5% *M. gaditana*, (iii) 5% *T. galbana*, (iv) 5% *A. platensis*, and (v) 5% of a microalgal blend containing 25% of each species. To determine the possible background from the media, cellophane sheets were placed on all six media without inoculating the strains, serving as an internal control. The experimental conditions are summarized in Fig. 1. Each experimental condition was conducted in triplicate to ensure reproducibility. For each replicate, ten individual plates were bulked together, and this process was repeated three times to generate three independent harvests. Incubation of all plates was carried out at 23 °C for 24 h.

After incubation, bacterial cells from the various growth conditions and internal controls were collected in 2 mL sterile phosphate-buffered saline (PBS, pH 7.2) and centrifuged at 10,000 × *g* for 20 min at 4 °C. The supernatants were then passed through 0.45 and 0.2 µm pore-size membrane filters (Merck Millipore, USA) to obtain only the ECPs. The protein concentration was determined using the Qubit Protein assay kits and the Qubit 2.0 fluorometer (Thermo Fisher Scientific, USA). Aliquots of the ECP samples were cultured on TSA plates with 1.5% NaCl and incubated for 24 h at 23 °C to confirm the absence of growth. The ECPs were stored at − 80 °C until further use.

### Hydrolytic-Enzyme Production

Caseinase, gelatinase, lipolytic, and amylolytic activities were assessed on agar plates containing 2% (w/v) skim milk (Pirinea, Spain), 1% (w/v) gelatin (Oxoid Ltd.), 1% (w/v) Tween-80 (Panreac, USA), and 4% (w/v) starch (Labkem, USA), respectively, following the method described by Chabrillón et al. [24]. Additionally, phytase, tannase, and cellulase activities were measured on agar plates (1.5% agar) containing 1% (w/v) Na-phytate (P-8810, Sigma), 2% (w/v) tannic acid (P-403040, Sigma), and 1% (w/v) carboxymethyl cellulose (CMC) (C-5678, Sigma), respectively, as described by Kumar et al. [25]. In each plate, 6 mm-diameter wells were cut, and 50 µL of two-fold serial dilutions of each ECP sample and internal controls was inoculated into the wells. The plates were incubated at 23 °C for 24–48 h. After incubation, the plates were examined for the presence of a clear zone surrounding the wells. Starch and cellulose hydrolysis was indicated by a clear zone around the wells after flooding the plates with Lugol’s iodine solution and Congo red solution (0.1% w/v), respectively. Negative controls (50 µL of PBS) and positive controls (50 µL of *Vibrio proteolyticus* cells at a concentration of 10^8^ cfu mL^−1^) were included [26]. The absence of a clear zone indicated the absence of activity. The minimum concentration of each activity was determined as the lowest ECP concentration with a clear zone around the well. Each ECP condition was tested in triplicate, and each experiment was performed in triplicate.

### Antagonistic Activity Against Fish Pathogens

The agar-well diffusion assay described by Mathabe et al. [27] and García-Márquez et al. [28] was used to evaluate antibacterial activity. Fish pathogenic bacterial strains *Vibrio harveyi* 16/00 [29] and *P. damselae* subsp. *piscicida* [30] were cultured on TSA plates with 1.5% NaCl at 23 °C for 24 h. *Tenacibaculum maritimum* (Spanish Type Culture Collection, CECT 4276) was cultured on *Flexibacter maritimus* medium (FMM) [31] plates supplemented with 1.5% agar at 28 °C for 48 h. Standardized cultures adjusted to an optical density at 600 nm (OD_600nm_) = 0.1 were spread evenly onto the surface of TSA or FMM plates using sterile swab sticks. To assess the activity of the ECPs, 50 µL of two-fold serial dilutions of each ECP sample and internal controls was inoculated into 6 mm-diameter wells made in the plates and incubated at 23 °C (*V. harveyi* and *P. damselae* subsp. *piscicida*) or 28 °C (*T. maritimum*) for 24–48 h. Negative controls (50 µL of TSB or FMM) and positive controls (50 µL of *V. proteolyticus* cells at a concentration of 10^8^ cfu mL^−1^) were included [26]. The presence or absence of an inhibition zone around each well determined the antibacterial activity. The minimum inhibitory concentration (MIC) was defined as the lowest ECP concentration that inhibited bacterial growth. Each ECP condition was tested in triplicate, and each experiment was performed in triplicate.

### Hemolytic Activity

The hemolytic activity of the ECPs was determined using Columbia agar plates containing 5% (w/v) sheep blood (Oxoid). For this, 50 µL of each ECP sample and internal controls was inoculated into 6 mm-diameter wells made in the plates and incubated at 23 °C for 24–48 h. The hemolytic activity of the ECPs was determined according to the signs of α-hemolysis (green zones around colonies), β-hemolysis (clear zones around colonies), or γ-hemolysis (no zones around colonies) on the plates [32].

In view of the enzymatic hydrolysis, antimicrobial, and hemolytic activities, we reduced the number of ECP conditions to focus on the most promising ECPs and continue evaluating their potential biotechnological application. Thus, 4 conditions were selected for further analysis.

### In Vitro Protein Hydrolysis Assay

Prior to carrying out the in vitro hydrolysis, a determination of the total alkaline protease activity of each one of the four ECPs selected was assessed. In order to understand how ECPs degrade microalgae biomass, in vitro, hydrolysis was carried out in 10 mL bioreactors, connected to a water circuit at 37 °C, under constant stirring. An amount of microalgae biomass was used that provided 80 mg of crude protein. This amount was suspended in 50 mM Tris HCl buffer pH 9.0, and the hydrolysis was started by adding a volume of ECPs that would provide 200 units of total alkaline protease activity, following a modification of the method described by Vizcaíno et al. [7]. In addition, control assays were carried out, which included microalgae biomass, but where ECPs were not added. The in vitro hydrolysis process was maintained for 120 min, and samples of the reaction mixture were withdrawn at different times (0, 15, 30, 60, 90, and 120 min). Each assay was performed in triplicate.

### Sequential Characterization of the In Vitro Hydrolysis

The protein hydrolysis of the different species of microalgae evaluated by the action of the ECPs was assessed through the sequential characterization of the products released to the reaction vessel, by polyacrylamide gel electrophoresis, using sodium dodecyl sulfate (SDS-PAGE), following the methodology described by Laemmli [33]. Previously, samples were diluted (1:1) in 0.125 M Tris HCl sample buffer, pH 6.8; 4% (w/v) SDS; 10% (v/v) β-mercaptoethanol; 20% (v/v) glycerol; 0.04% (w/v) of bromophenol blue, and boiled during a pulse, with the aim of stopping the enzymatic reaction. Furthermore, the degree of protein hydrolysis was quantified by calculating the protein degradation coefficient (CPD), according to Alarcón et al. [34].

The amino acids released during the in vitro hydrolysis process were determined by the O-phthaldialdehyde method [35], using L-leucine as standard. Undigested protein was discarded by precipitation with trichloroacetic acid (1:1). Blank assays were carried out, which made it possible to determine the net release of amino acids attributable to the action of the ECPs. The results were expressed as g of released amino acids (g L-leucine equivalents) per 100 g^−1^ of protein.

The quantification of the content of soluble protein released to the bioreactor during in vitro enzymatic hydrolysis was carried out by the method described by Bradford [36], using bovine serum albumin as a standard. Finally, the reducing sugars released during the in vitro hydrolysis process were quantified following the methodology described by Miller [37], using dinitrosalicylic acid (DNS). All assays were carried out in triplicate. In addition, blank tests were carried out, without ECPs, for each one of the microalgae analyzed.

### Animal Maintenance and Ethics

Juveniles of gilthead seabream (*S. aurata*) were obtained from a commercial source (CUPIBAR, Chiclana de la Frontera, Cádiz) and acclimated to the indoor experimental facilities at the *Servicios Centrales de Investigacion en Cultivos Marinos* (SCI-CM, CASEM, University of Cadiz, Puerto Real, Cadiz, Spain; Spanish Operational Code REGA ES11028000312) in an open circulatory system with seawater in controlled conditions of salinity (37 ppt), temperature (19 °C), and under natural photoperiod at our latitude (36°31′45″ N, 6°11′31″ W, from October to December 2022). Experimental procedures were done following the guidelines for experimental procedures in animal research of the Ethics and Animal Welfare Committee of the University of Cadiz, according to the principles published in the European Animal Directive (2010/63/EU) and Spanish laws (Royal Decree RD53/2013) for the protection of animals used in scientific experiments. The Ethical Committee from the Autonomous Andalusian Government also approved the experiments (Junta de Andalucía reference number 3/11/2021/172).

### Experimental Diets

ECP-nanoparticles were obtained following a modification of the methodology described by Fernández-Díaz et al. [38]. Briefly, low molecular weight chitosan (CS) (Brookfield viscosity 20,000 cps) (Sigma-Aldrich, USA) was dissolved in 0.4% glacial acetic acid providing a final concentration of 1 mg chitosan mL^−1^. Then, pH was adjusted to 4.7 using NaOH, and the solution was kept at 4 °C until use. For the nanoparticle preparation, sodium-tripolyphosphate (TPP) was dissolved in the ECP solution to reach a final concentration of 0.75 mg mL^−1^ (pH 7). This solution was kept at 4 °C, and then, it was rapidly added to the chitosan solution previously heated at 45 °C (1:3, v:v) under continuous agitation (300 rpm). The formed nanoparticles were allowed to stabilize for 15 min at 4 °C and then filtered for the elaboration of experimental aquafeeds.

Five experimental feeds were elaborated: (i) a control diet (CT) mimicking the ingredient composition of commercial diets for gilthead seabream, including 10% fishmeal and 7% fish oil, (ii) a diet supplemented with 5% of a blend of microalgae (25% *C. vulgaris*, 25% *A. platensis*, and 50% *M. gaditana*) for replacing terrestrial plant protein (MICROALGAE), (iii) the microalgae-supplemented diet enriched with the ECP-nanoparticles (10 mL kg^−1^) incorporated to the ingredient mixture before the pellet extrusion (E-10 M), (iv) the microalgae-supplemented diet enriched with the ECP solution (5 mL kg^−1^) applied to the feed pellets after extrusion by using a vacuum fat coater (E-5 V), and (v) the microalgae-supplemented diet enriched with the ECP solution (10 mL kg^−1^) applied to the feed pellets after extrusion by using a vacuum fat coater (E-10 V). Diets were produced with a diameter of 2 and 3 mm by the University of Almeria, Spain. Briefly, all ingredients were mixed in a 10 L mixer and ground with a hammer mill (UPZ 100, Hosokawa-Alpine, Augsburg, Germany) to 0.5 mm. The diets were cold-extruded in a single-screw extruder (Miltenz 51SP, JS Conwell Ltd., New Zealand), fitted with 2 or 3-mm die holes. The extruder barrel consisted of four sections, and the temperature profile in each segment (from inlet to outlet) was 40, 40, 45, and 45 °C, respectively. The pellets were dried after extrusion at 27 °C using a drying chamber (Airfrio, Almería, Spain) and cooled at ambient temperature. Vacuum fat coating was done on the following day in a Pegasus PG-10VC LAB vacuum coater (Dinnissen, Sevenum, The Netherlands). Ingredients and proximate composition of the experimental diets are shown in Supplementary Table 1.

### Feeding Protocol and Sampling Procedures

Five different dietary treatments, corresponding to the five experimental diets, were applied over a period of 8 weeks, using a total of 375 specimens with an initial mean body mass of 18.05 ± 0.02 g. Fish were individually weighed and randomly distributed in 15 tanks of 400 L capacity (*n* = 25 fish/tank) adjusted to a total volume of 180 L (initial stocking density 2.50 ± 0.02 kg/m^3^) in the SCI-CM and were kept during the whole experimental period in an open circulatory system as described above. Prior to offering the experimental diets, fish were acclimated to the experimental units for 10 days, and then, feeding was supplied until apparent satiety (ad libitum), ensuring that the amount offered in each experimental unit was fully ingested. The feeding test was carried out blindly, in such a way that the five aquafeeds were labeled with different colors but with no reference to their composition, eliminating any source of subjectivity when feeding the animals. No mortality was recorded in any experimental group.

At the end of the feeding trial, a final sampling was done, in which 12 overnight fasted specimens from each experimental diet (4 fish per tank) were randomly selected, deeply anesthetized with a lethal dose of 2-phenoxyethanol, and then individually weighed and measured. For plasma samples, blood was drawn from the caudal vessels with heparinized syringes and centrifuged at 13,000 × *g* for 3 min at 4 °C. Livers were removed and weighed from each specimen. Both plasma samples and liver biopsies were snap-frozen in liquid nitrogen and stored at − 80 °C until further biochemical analysis. The complete intestine was also removed for length measurement from the pyloric caeca to the rectum. For the digestive enzymatic analysis, the intestine samples were snap-frozen in liquid nitrogen and stored at − 80 °C until their use. Finally, the remaining fish of each experimental group were also weighted and measured to obtain the growth performance and biometric parameters described below for the total of animals assayed.

### Growth Performance and Biometric Parameters

The growth parameters evaluated were (i) specific growth rate (SGR) = 100 × (ln final body weight − ln initial body weight)/days; (ii) weight gain (WG) = 100 × (body weight increase)/initial body weight; (iii) feed efficiency (FE) = weight gain/total feed intake; and (iv) condition factor (*K*) = (100 × body weight)/fork length^3^.

Organosomatic indexes are the ratio of tissue to body weight or fork length, and they were calculated with the following equations (i) hepatosomatic index (HSI) = (100 × liver weight)/fish weight and (ii) intestine length index (ILI) = (100 × Li)/Lb, where Li and Lb are the intestine and fork body length, respectively.

### Metabolic Response

For plasma analyses, commercial kits (SpinReact SA, St. Esteve d’en Bas, Girona, Spain) were used, with reactions adapted to 96-well microplates. The metabolites assayed include levels of glucose (Glucose-HK Ref. 13 1,001,200), lactate (Lactate Ref. 1,001,330), cholesterol (Cholesterol-LQ Ref. 41,021), and triglycerides (TAG Ref. 1,001,311). The total protein concentration was determined using the BCA kit (BCA™ Protein assay kit, Pierce, Rockford, USA). Cortisol levels were measured with the Cortisol Enzyme Immunoassay Kit (Arbor Assays, K003-H1W) following the manufacturer’s indications.

For liver analyses, frozen biopsies were mechanically homogenized in 7.5 volumes of ice-cold 0.6N perchloric acid. Then, samples were neutralized using 1 M KCO_3_. An aliquot was taken for triglyceride analysis. After centrifugation (30 min, 3220 × g, 4 °C), the supernatants were used to determine stored metabolites. Tissue triglycerides and lactate levels were determined with a commercial kit (SpinReact, see above). Tissue glycogen concentration was quantified using the method described by Keppler and Decker [39], where glucose obtained after glycogen breakdown with amyloglucosidase (Sigma-Aldrich, Ref. A7420) was determined with a commercial kit (SpinReact) as described before.

All assays were performed with a PowerWave™ 340 microplate spectrophotometer (BioTek Instruments, Winooski, VT, USA), controlled by Gen5 Software for Microsoft® Windows.

### Digestive Enzyme Analysis

Prior to the digestive enzymatic analysis, intestine samples were homogenized in distilled water (4 °C) until obtaining a concentration of 0.5 g tissue mL^−1^ and then were centrifuged (16,000 × *g*, 12 min, 4 °C), and the supernatants obtained were stored at − 20 °C until further use. Total alkaline protease enzyme activity was determined according to Alarcón et al. [40], using 5 g L^−1^ of casein in 50 mM Tris–HCl (pH 9.0) as substrate. One unit of total alkaline protease activity was defined as the amount of enzyme that released 1 µg of tyrosine per min in the reaction mixture, considering an extinction coefficient of 0.008 µg^−1^ mL^−1^ cm^−1^ for tyrosine, measured at 280 nm wavelength. Trypsin and chymotrypsin enzymatic activities were determined according to the methodology described by Erlanger et al. [41] and DelMar et al. [42], respectively, using 0.5 mM BAPNA (N-α-benzoyl-DL-arginine-4-nitroanilide) and 0.2 mM SAPNA (N-succinyl-(Ala)_2_-Pro-Phe-p-nitroanilide), in 50 mM Tris–HCl buffer, 20 mM CaCl_2_, pH 8.5, as substrate, respectively. Leucine aminopeptidase activity was determined spectrophotometrically following the procedure described by Pfleiderer [43]. For these three enzymatic activities, a unit of enzymatic activity (U) was defined as the amount of enzyme that releases 1 µmol of p-nitroanilide (pNA) per minute, considering the extinction coefficient to be 8800 M cm^−1^, measured spectrophotometrically at 405 nm. Alkaline phosphatase activity was measured in supernatants obtained from fish intestines using 450 mM p-nitrophenyl phosphate in 1 M diethanolamine, 1 mM MgCl_2_ buffer, pH 9.5, according to the method described by Bergmeyer [44]. For alkaline phosphatase, one unit of activity was defined as the amount of enzyme that released 1 µg of nitrophenyl per minute considering an extinction coefficient of 17,800 M cm^−1^ for p-nitrophenol, also measured at 405 nm. All assays were performed in triplicate.

### Statistical Analysis

Statistical analyses were conducted using IBM SPSS Statistics 22.0. Normality and homogeneity of variance of the data were determined by using Shapiro–Wilk and Levene’s tests, respectively. Differences were statistically analyzed by one-way analysis of variance (ANOVA) with Tukey and Games-Howell post hoc tests when statistical requirements were fulfilled. Non-normally distributed data were analyzed by the non-parametric Kruskal–Wallis test, followed by a multiple comparison test. Statistical significance was set for *p* ≤ 0.05.

## Results

The two bacterial strains did not exhibit any growth when cultured in media supplemented with *Tisochrysis galbana* at 5% (w/w). As a result, this particular medium was excluded from further use in obtaining extracellular products (ECPs).

### Hydrolytic, Antimicrobial, and Hemolytic Activity of the ECPs

The hydrolytic, antimicrobial, and hemolytic activities of the ECPs were evaluated, and the results are summarized in Table 1. None of the ECPs showed activity in terms of starch, lipase, phytase, or tannase hydrolysis. Enzymatic activity assays revealed that all ECPs except TSA 169 hydrolyze gelatin, and all ECPs except TSA 169 and CHL 169 exhibited milk hydrolysis. Interestingly, four conditions (MICRO 169, ATH 169, MIX 169, and MICRO 216) exhibited cellulose hydrolysis activity.

**Table 1:** Hydrolytic, antimicrobial, and hemolytic activities produced by ECP samples extracted from different conditions

	UMA-169					UMA-216				
	TSA 169	CHL 169	MICRO 169	ATH 169	MIX 169	TSA 216	CHL 216	MICRO 216	ATH 216	MIX 216
Hydrolytic activity										
Amylase	ND	ND	ND	ND	ND	ND	ND	ND	ND	ND
Gelatinase	ND	8.3 ± 0.3	10.1 ± 0.5	9.0 ± 0.3	12.0 ± 0.9	7.2 ± 0.4	6.7 ± 0.1	17.7 ± 1.1	6.9 ± 0.2	10.0 ± 0.2
Caseinase	ND	ND	20.2 ± 0.8	71.8 ± 2.2	47.9 ± 1.3	114.5 ± 3.2	53.5 ± 2.8	35.4 ± 2.0	110.5 ± 4.1	20.1 ± 0.8
Lipase	ND	ND	ND	ND	ND	ND	ND	ND	ND	ND
Phytase	ND	ND	ND	ND	ND	ND	ND	ND	ND	ND
Tannase	ND	ND	ND	ND	ND	ND	ND	ND	ND	ND
Cellulase	ND	ND	646 ± 7.9	574 ± 9.8	383 ± 5.2	ND	ND	567 ± 8.9	ND	ND
Antimicrobial activity										
*V. harveyi*	291.0 ± 3.1	ND	323.0 ± 2.9	ND	766.0 ± 8.4	458.0 ± 6.5	428.0 ± 2.4	1134.0 ± 9.8	442.0 ± 5.5	321.0 ± 2.7
*P. damselae* subsp. *piscicida*	145.5 ± 2.4	266.0 ± 1.8	323.0 ± 2.9	287.0 ± 2.4	383.0 ± 3.2	114.5 ± 3.7	428.0 ± 2.4	567.0 ± 6.5	221.0 ± 3.2	321.0 ± 2.7
*T. maritimum*	ND	ND	ND	ND	ND	542	ND	ND	ND	ND
Virulence factors										
*Hemolysis*	γ	γ	γ	γ	γ	γ	γ	γ	γ	γ

The results are expressed mean ± SD of the minimum concentration in the ECPs for each activity (µg protein mL^−1^)

*ND* not detected

As for the antimicrobial activity of the ECPs, all tested conditions were capable of inhibiting the growth of *P. damselae* subsp. *piscicida*, and only two conditions (CHL 169 and ATH 169) did not exhibit inhibitory effects against *V. harveyi*. Furthermore, *T. maritimum* was inhibited only by TSA 216, which was the only condition that demonstrated inhibitory activity against all three tested pathogens.

To assess the hemolytic activity of the ECPs, blood agar plates were used. All ECP conditions showed γ-hemolytic activity, indicating no hemolysis. Notably, the internal controls did not exhibit any hydrolytic enzyme activity, antimicrobial effects, or hemolytic activity.

Based on the hydrolytic, antimicrobial, and hemolytic activities of the postbiotics, we selected MICRO 169, TSA 216, CHL 216, and MICRO 216 for in vitro microalgae hydrolysis.

### In Vitro Hydrolysis

#### Sequential Characterization of the In Vitro Protein Degradation

**Fig. 2:**
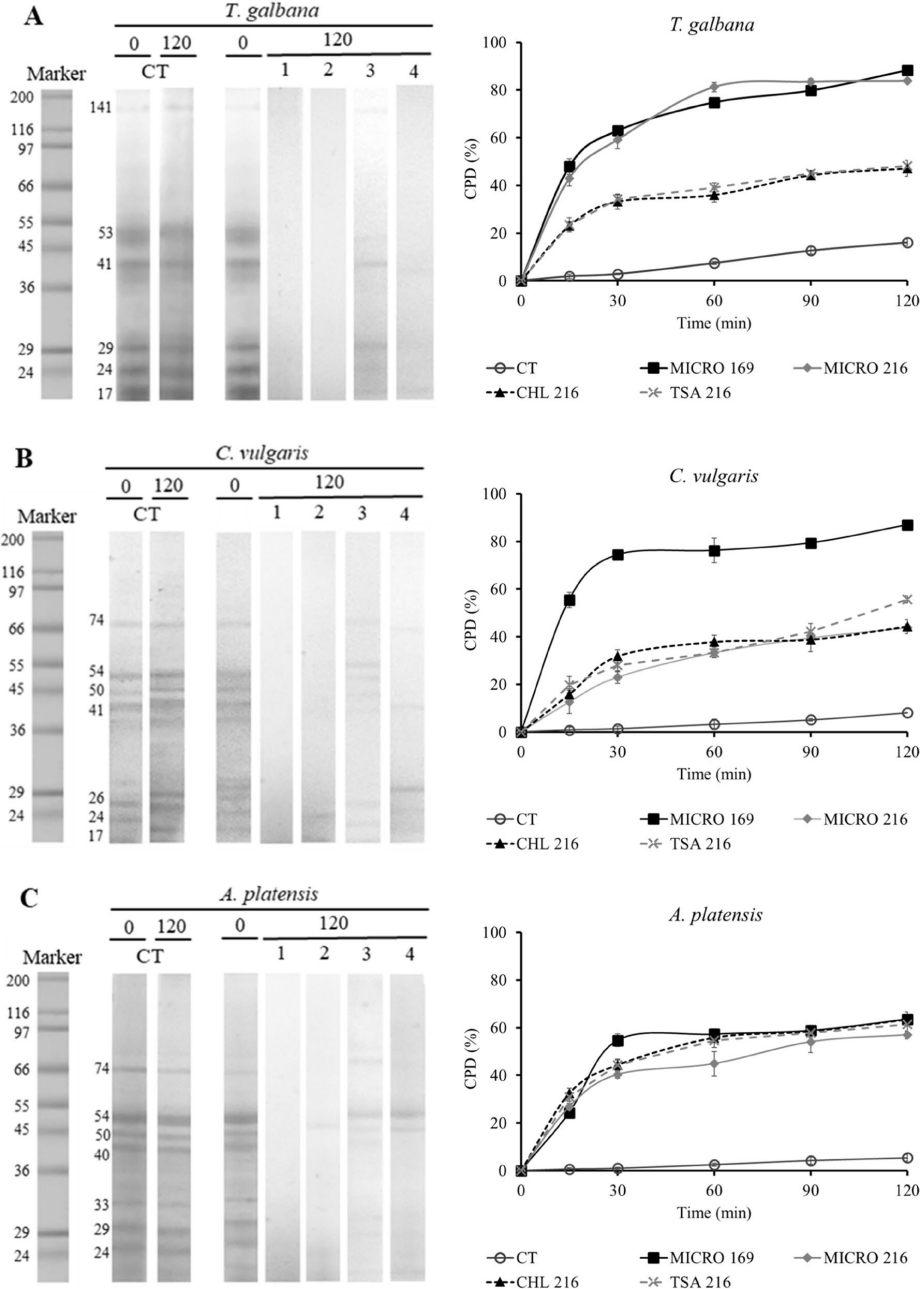

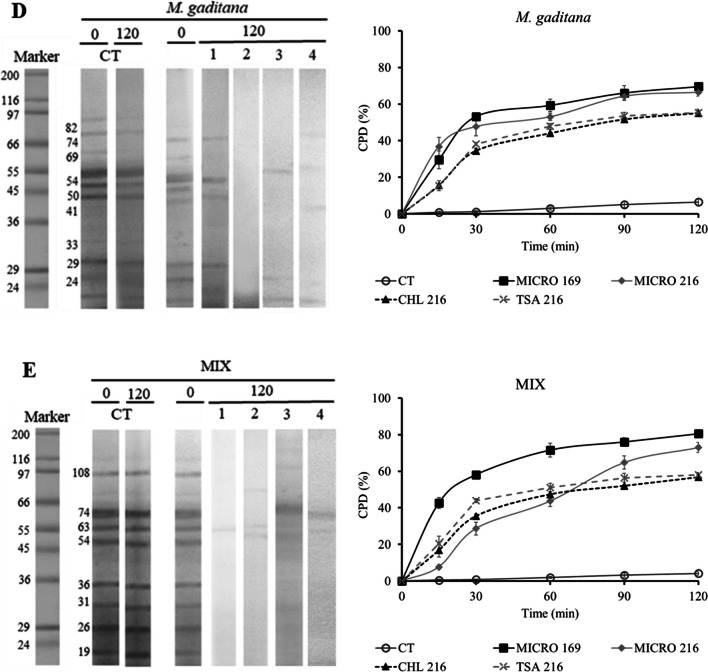
Time-course of in vitro proteolysis and coefficient of protein degradation (CPD) of *T. galbana*, *C. vulgaris*, *A. platensis*, *M. gaditana*, and the mix of microalgae (**A**–**E**, respectively) by the action of the different ECPs evaluated. CT corresponds to blank assays performed in the absence of ECPs. The values located on the lanes indicate the ECP used (1: MICRO 169; 2: MICRO 216; 3: CHL 216; 4: TSA 216). The molecular weights of the main protein fractions are indicated to the left of the marker and the initial CT time lane

The total proteolytic activity measured in MICRO 169, MICRO 216, CHL 216, and TSA 216 was 946.9 ± 41.8, 853.2 ± 32.3, 242.4 ± 30.0, and 363.9 ± 8.6 U mL^−1^, respectively. Figure 2 and Supplementary Fig. 1 depict the progression of protein in vitro hydrolysis across various microalgae species under examination by the ECPs after a 120-min assay. Notably, protein degradation was prevalent in most fractions after this period, especially with MICRO 169 and MICRO 216. The coefficient of protein degradation (CPD), calculated from optical density data in SDS-PAGE electrophoresis gels, revealed rapid protein degradation within the first 30 min, followed by stabilization. MICRO 169 and MICRO 216 consistently demonstrated the highest protein degradation percentages, particularly with *T. galbana* and *C. vulgaris*, where values surpassed 80%. For *M. gaditana* and the mix of microalgae, MICRO 169 and MICRO 216 still yielded higher results compared to the other conditions, with protein degradation percentages exceeding 65% and 70%, respectively.

#### Quantification of the Amino Acid Released

**Fig. 3:**
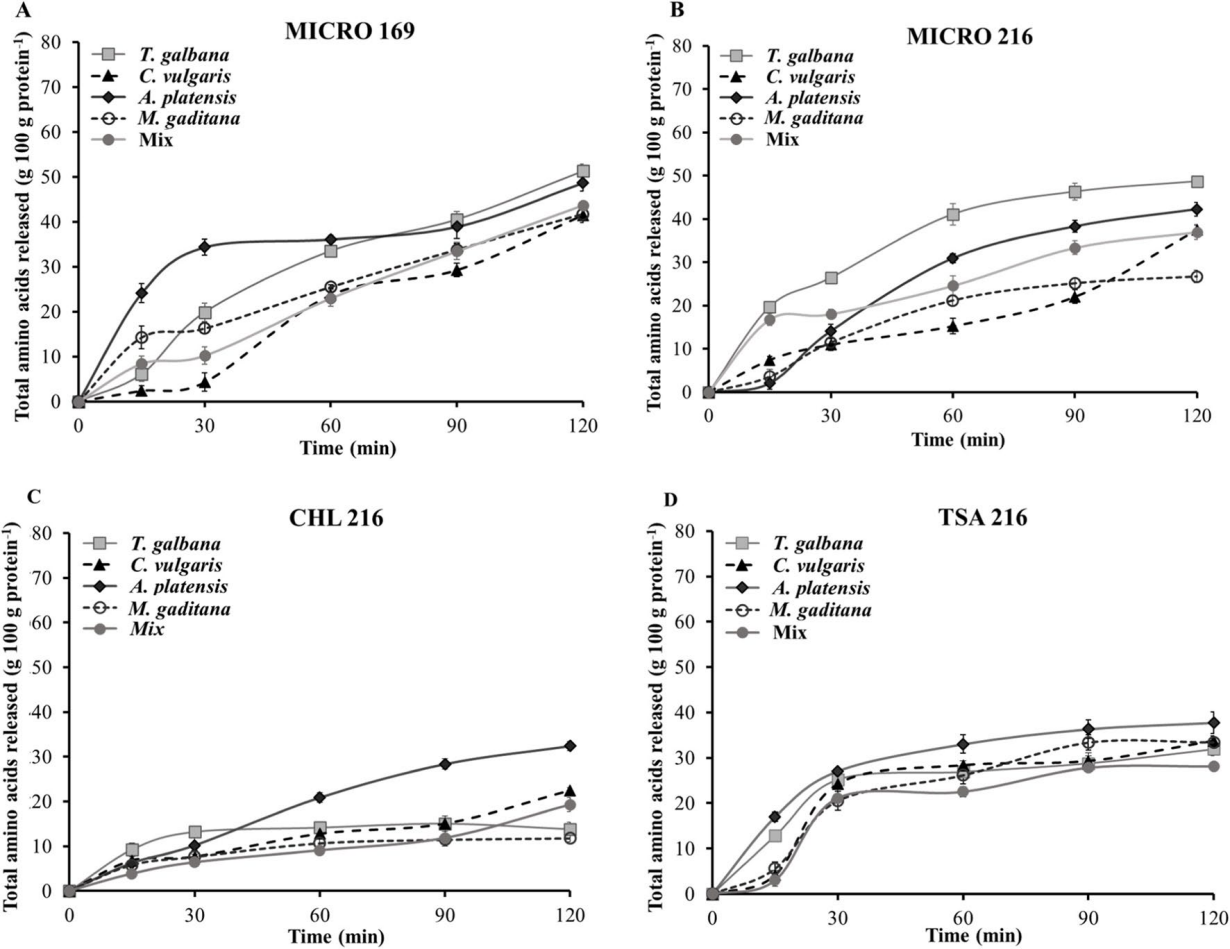
Quantification of amino acid released during in vitro hydrolysis assay by the different ECPs (**A** MICRO 169; **B** MICRO 216; **C** CHL 216; **D** TSA 216). Values represent the mean ± SD of triplicate determinations

Figure 3 shows the values of amino acid release during the in vitro hydrolysis process by different ECPs. A continuous release of amino acids was observed throughout the assay, more pronounced with MICRO 169 and MICRO 216 conditions, where values higher than 40 g of amino acids per 100 g of protein in the case of MICRO 169 were observed regardless of the species of microalgae used and values between 25 and 51 g of amino acids released per g of protein in the case of the assays carried out with the ECP MICRO 216. Results obtained for CHL 216 and TSA 216 showed a similar trend, but the final values were lower compared to the other two ECPs.

#### Quantification of Soluble Protein Concentration

**Fig. 4:**
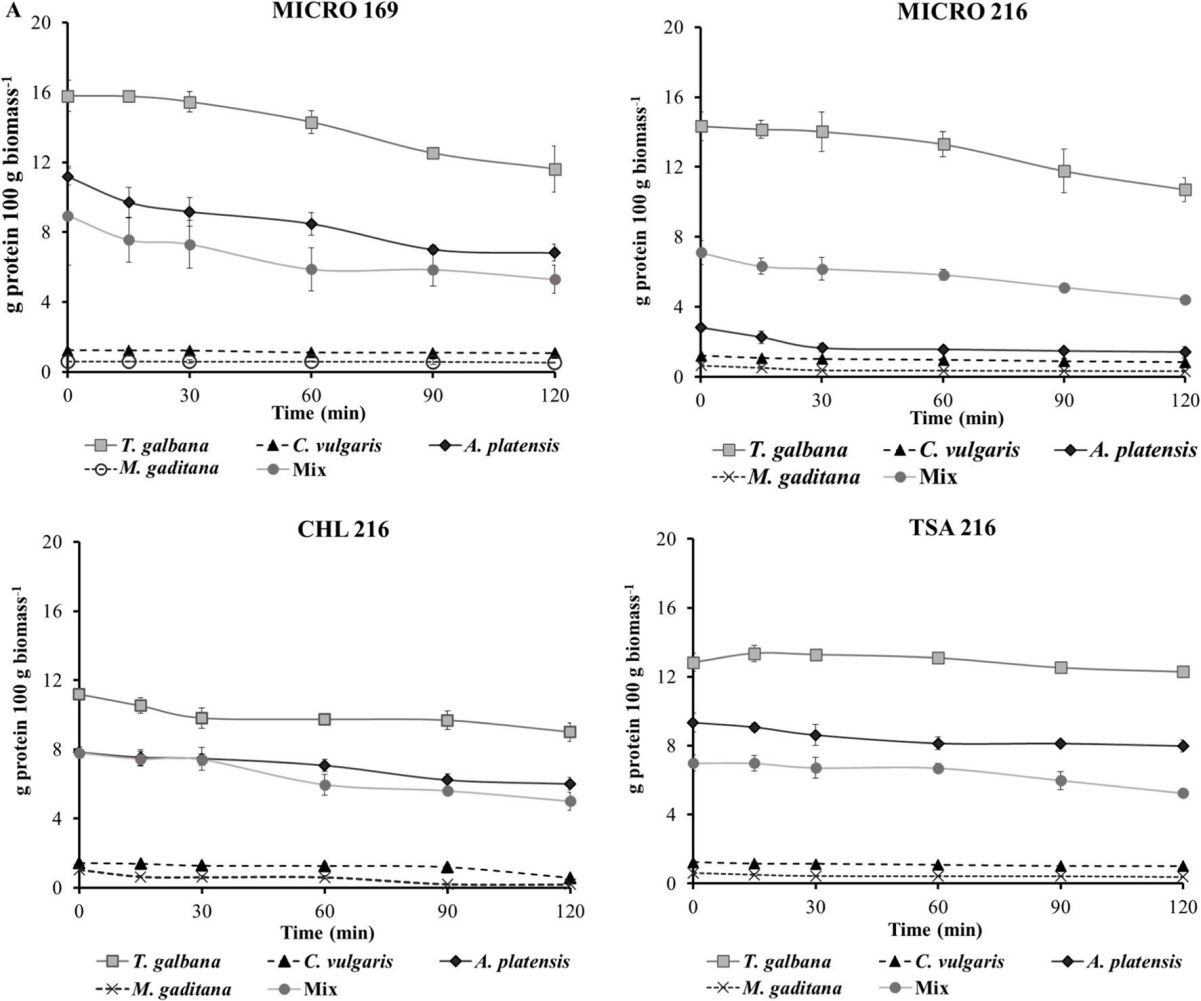
Quantification of the release of soluble protein throughout the in vitro hydrolysis process by the action of the different ECPs (MICRO 169, MICRO 216, CHL 216, TSA 216). Values represent the mean ± SD of triplicate determinations

The quantification of soluble protein concentration during in vitro hydrolysis assays is presented in Fig. 4. In general, *T. galbana* exhibited the highest concentration of soluble protein, followed by *A. platensis*, while *M. gaditana* and *C. vulgaris* showed the lowest values. A decline in soluble protein concentration was evident throughout the hydrolysis, particularly in *T. galbana* and *A. platensis* when hydrolyzed by MICRO 169, registering a decrease of over 4 g of protein per 100 g of biomass. A similar decrease in protein concentration was observed with MICRO 216, notably in *T. galbana* and *A. platensis*, with a decrease of 3.6 and 1.4 g of protein per 100 g of biomass, respectively.

#### Quantification of the Release of Reducing Sugars

**Fig. 5:**
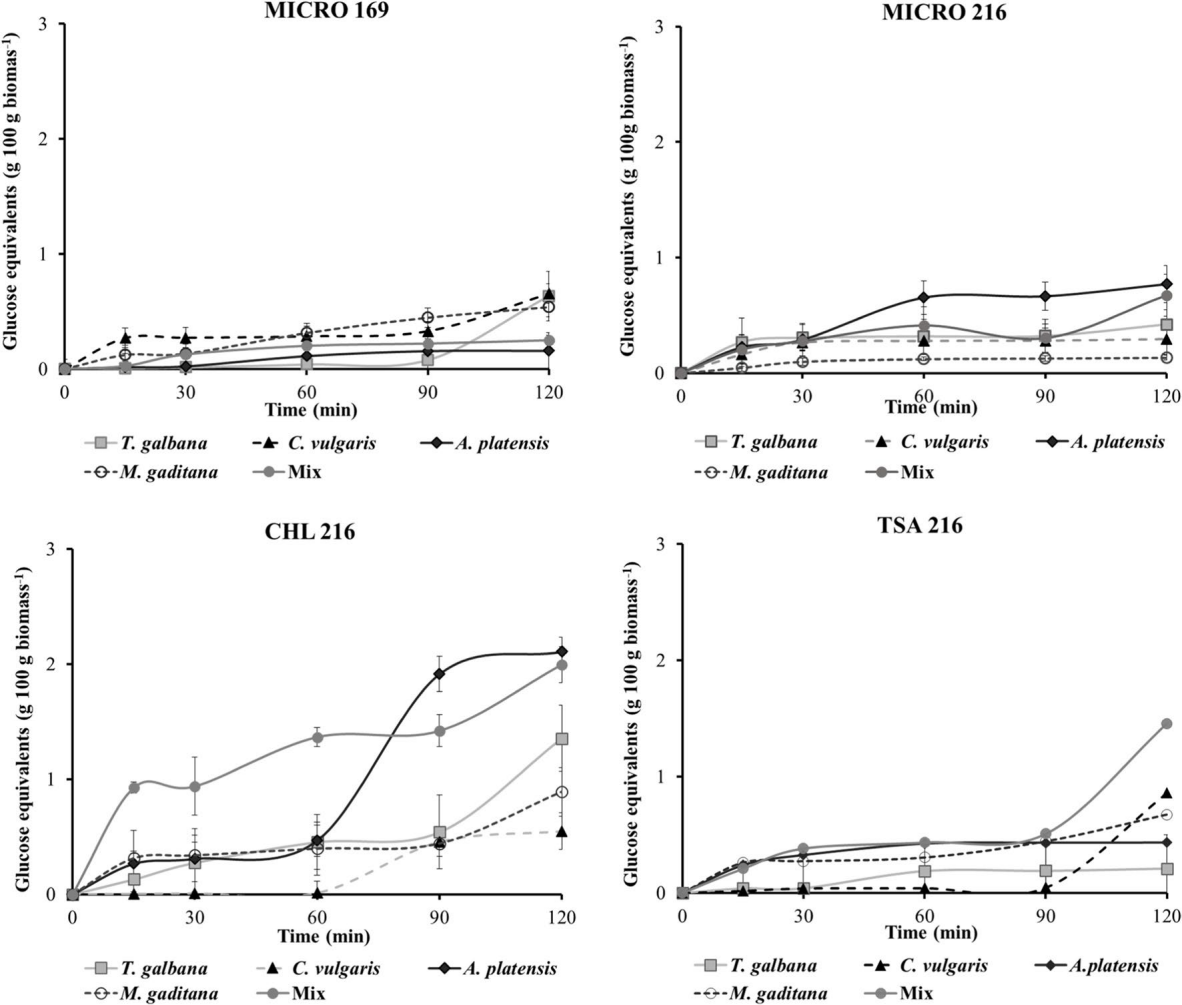
Quantification of reducing sugars released during the in vitro assay by the action of ECPs (MICRO 169, MICRO 216, CHL 216, TSA 216). Values represent the mean ± SD of triplicate determinations

Figure 5 illustrates the release of reducing sugars during the 120-min in vitro hydrolysis. Overall, minimal sugar release was observed due to ECP action, with values consistently below 2.5 g of glucose equivalents per 100 g of biomass. Higher values were noted in assays with TSA 216 and CHL 216, while MICRO 169 and MICRO 216 yielded lower values, with less than 1 g of glucose equivalents released per 100 g of biomass across all microalgae evaluated.

### Feeding Trial

#### Growth Performance and Nutrient Utilization

No mortality occurred during the experiment. As shown in Table 2, no differences were found regarding growth parameters and somatic indices between the experimental groups.

**Table 2:** Growth performance and somatic indexes of gilthead seabream juveniles fed the experimental diets

Parameters	CT	MICROALGAE	E10-M	E5-V	E10-V	*p*^a^
Initial body weight (g)	18.00 ± 0.03	18.08 ± 0.01	18.01 ± 0.02	18.11 ± 0.03	18.03 ± 0.03	0.0595
Final body weight (g)	25.78 ± 0.68	25.54 ± 0.59	26.30 ± 0.59	24.96 ± 0.58	24.93 ± 0.20	0.4178
Final length (cm)	11.37 ± 0.11	11.33 ± 0.05	11.51 ± 0.16	11.48 ± 0.09	11.37 ± 0.06	0.6917
K^b^	1.70 ± 0.02	1.74 ± 0.03	1.70 ± 0.02	1.72 ± 0.02	1.78 ± 0.03	0.0644
SGR^c^	0.64 ± 0.05	0.62 ± 0.04	0.68 ± 0.04	0.57 ± 0.04	0.58 ± 0.01	0.3661
WG^d^	43.20 ± 3.91	41.30 ± 3.38	46.00 ± 3.40	37.84 ± 3.41	38.26 ± 0.88	0.3856
FE^e^	0.31 ± 0.02	0.34 ± 0.03	0.39 ± 0.03	0.35 ± 0.06	0.33 ± 0.03	0.7019
FI^f^	23.97 ± 1.21	21.89 ± 1.41	21.35 ± 1.32	19.99 ± 2.16	21.23 ± 1.66	0.2124
HSI^g^	1.72 ± 0.07	1.85 ± 0.22	2.13 ± 0.15	2.13 ± 0.13	2.26 ± 0.20	0.1364
ILI^h^	62.63 ± 3.31	67.70 ± 3.27	57.01 ± 3.92	62.56 ± 3.50	65.08 ± 3.88	0.3132

Dietary codes: CT: diet control without ECP and microalgae. MICROALGAE: diet supplemented with 5% of a blend of microalgae. E-10 M: microalgae-supplemented diet enriched with the ECP-nanoparticles (10 mL kg^−1^). E-5 V: the microalgae-supplemented diet enriched with the ECP solution (5 mL kg^−1^). E-10 V: the microalgae-supplemented diet enriched with the ECP solution (10 mL kg^−1^). The results of growth performance are expressed as the mean ± SEM of triplicate tanks. Data on somatic indices are the mean ± SEM of 12 fish

^a^Values resulting from one-way analysis of variance

^b^Condition factor (*K*) = (100 × body weight)/fork length^3^

^c^Specific growth rate (SGR) = 100 × (ln final body weight − ln initial body weight)/days

^d^Weight gain (WG, %) = 100 × (body weight increase)/initial body weight

^e^Feed efficiency (FE) = weight gain/total feed intake

^f^Feed intake (FI): g of aquafeed ingested/fish in the total period of the feeding trial

^g^Hepatosomatic index (HIS) = (100 × liver weight)/fish weight

^h^Intestine length index (ILI) = (100 × intestine length)/fork length

#### Metabolic Response

Results on plasma and liver metabolites are shown in Table 3. The results indicate that none of the treatments significantly affected the plasmatic and hepatic metabolism, or circulant levels of cortisol, of the fish when compared to the control group.

**Table 3:** Metabolic and hormonal parameters measured in plasma and liver of gilthead seabream juveniles fed the experimental diets

Parameters	CT	MICROALGAE	E10-M	E5-V	E10-V	*p*^a^
Plasma						
Glucose (mM)	3.96 ± 0.21	3.99 ± 0.17	4.61 ± 0.40	4.02 ± 0.15	4.06 ± 0.27	0.3731
Lactate (mM)	1.59 ± 0.14	1.27 ± 0.13	1.63 ± 0.14	1.69 ± 0.09	1.76 ± 0.21	0.1741
Triglycerides (mM)	3.26 ± 0.42	4.19 ± 0.47	3.94 ± 0.33	3.99 ± 0.57	3.09 ± 0.24	0.2552
Cholesterol (mM)	3.75 ± 0.18	3.92 ± 0.32	4.33 ± 0.20	3.84 ± 0.20	3.95 ± 0.25	0.4872
Total proteins (mg mL^−1^)	36.55 ± 1.35	37.90 ± 1.55	37.20 ± 0.52	37.71 ± 1.17	36.89 ± 1.49	0.9396
Cortisol (ng mL^−1^)	3.39 ± 0.89	2.62 ± 0.49	3.59 ± 0.92	4.06 ± 1.26	3.20 ± 0.52	0.9209
Liver						
Glycogen (mM g^−1^ FW)	65.56 ± 3.42	69.32 ± 4.51	67.69 ± 2.46	71.49 ± 3.71	68.43 ± 5.86	0.8932
Glucose (mM g^−1^ FW)	12.90 ± 0.88	12.42 ± 0.87	12.81 ± 0.69	11.62 ± 0.53	11.38 ± 1.14	0.6222
Lactate (mM g^−1^ FW)	0.67 ± 0.04	0.55 ± 0.06	0.58 ± 0.08	0.65 ± 0.06	0.56 ± 0.05	0.4846
Triglycerides (mM g^−1^ FW)	72.47 ± 6.29	67.01 ± 4.75	57.10 ± 5.40	61.40 ± 4.29	73.36 ± 5.18	0.1411

Dietary codes: CT: diet control without ECP and microalgae. MICROALGAE: diet supplemented with 5% of a blend of microalgae. E-10 M: microalgae-supplemented diet enriched with the ECP-nanoparticles (10 mL kg^−1^). E-5 V: the microalgae-supplemented diet enriched with the ECP solution (5 mL kg^−1^). E-10 V: the microalgae-supplemented diet enriched with the ECP solution (10 mL kg^−1^). The results on metabolism are expressed as the mean ± SEM of 12 fish per experimental group

^a^Values resulting from one-way analysis of variance

#### Digestive Enzyme Activities

In general, feed supplementation with microalgae and ECPs increased significantly the enzymatic activities measured in the intestinal extracts compared to the control group (Table 4). The general trend observed in the different enzymatic activities of pancreatic secretion (trypsin, chymotrypsin, and total alkaline protease) was similar in all cases, with an increase of activity in treatments containing microalgae or ECP, particularly the E10-M group showed the highest values. In the case of the leucine aminopeptidase and alkaline phosphatase activities, the E10-M and E10-V groups showed significant differences compared to the control, while the E5-V group evidenced an increasing trend, but without significant differences.

**Table 4:** Digestive enzyme activities (U g^−1^ tissue) measured in intestinal extracts of gilthead seabream juveniles fed the experimental diets

	CT	MICROALGAE	E10-M	E5-V	E10-V	*p*^1^
Trypsin	0.20 ± 0.02^a^	0.26 ± 0.02^b^	0.38 ± 0.03^c^	0.27 ± 0.02^b^	0.25 ± 0.22^b^	0.0017
Chymotrypsin	0.31 ± 0.04^a^	0.54 ± 0.08^b^	0.82 ± 0.08^c^	0.42 ± 0.07^b^	0.45 ± 0.054^b^	0.0084
Total alkaline protease	271.97 ± 64.25^a^	327.63 ± 76.42^ab^	429.34 ± 73.45^b^	346.76 ± 61.11^ab^	400.15 ± 65.95^ab^	0.0129
Leucine aminopeptidase	0.11 ± 0.02^a^	0.14 ± 0.02^ab^	0.22 ± 0.03^b^	0.15 ± 0.02^ab^	0.16 ± 0.02^b^	0.0392
Alkaline phosphatase	4.53 ± 0.62^a^	4.61 ± 0.69^a^	5.56 ± 0.44^b^	5.09 ± 0.48^ab^	5.55 ± 0.69^b^	0.0477

Dietary codes: CT: diet control without ECP and microalgae. MICROALGAE: diet supplemented with 5% of a blend of microalgae. E-10 M: microalgae-supplemented diet enriched with the ECP-nanoparticles (10 mL kg^−1^). E-5 V: the microalgae-supplemented diet enriched with the ECP solution (5 mL kg^−1^). E-10 V: the microalgae-supplemented diet enriched with the ECP solution (10 mL kg^−1^). Values are mean ± SD

^1^Values resulting from one-way analysis of variance. Values in the same row with different lowercase superscript indicate significant differences owing to dietary treatments (*p* < 0.05)

## Discussion

The choice of culture medium profoundly influences bacterial metabolism and, consequently, the composition of their extracellular products (ECPs). This study focuses on exploiting microalgae-containing media to induce specific bacterial enzymatic activities that enhance the digestion and utilization of microalgae in fish feeds. This approach aligns with our broader goal of optimizing bacterial processes for the production of beneficial compounds in aquaculture.

To explore the potential of microalgae as a nutrient source, we employed various microalgae species, namely, *Tisochrysis galbana*, *Microchloropsis gaditana*, *Chlorella vulgaris*, *Arthrospira platensis*, and a mixture of the four microalgae, to grow two *Bacillus pumilus* strains (UMA 169 and UMA 216) and obtain their ECPs. Notably, media containing *T. galbana* did not support the growth of the assayed bacterial strains, preventing the acquisition of their ECPs. Therefore, careful selection of suitable microalgae species is essential to ensure consistent and reliable bacterial cultivation for different biotechnological purposes.

Probiotic derivatives include interesting enzymes that can improve the digestion and absorption of various energy sources and hydrolyze anti-nutritional factors present in aquafeeds [45]. Incorporating enzyme-producing probiotic derivatives into aquafeeds has the potential to enhance feed utilization in farmed species. The assessment of enzymatic activity revealed proteolytic activity in most of the assayed ECPs, with the ability to hydrolyze gelatin and casein in all ECPs obtained from UMA 169 and UMA 216, except TSA 169 and CHL 169 conditions. This highlights the potential of these postbiotics in degrading proteins. Previous studies have shown that the addition of pre-digested protein improves growth performance, nutrient utilization, intestinal microbiota, and immune response in fish [46–48].

Furthermore, we found that four postbiotic conditions (MICRO 169, ATH 169, MIX 169, and MICRO 216) exhibited cellulase activity. Cellulose is a component of the microalgae cell wall and can affect the digestibility of microalgae in fish diets [49]. Enhancing the digestibility of microalgae biomass is crucial, and some studies have shown that exogenous cellulase promotes growth and increases cellulase, amylase, and protease activity in fish [50].

Postbiotics could offer potential solutions to the emergence of multidrug-resistant bacteria due to the widespread use of antibiotics. All of the postbiotics tested in our study inhibited the growth of *P. damselae* subsp. *piscicida*, a major pathogen in aquaculture, whereas only two conditions (CHL 169 and ATH 169) did not inhibit *V. harveyi*, another common pathogen. Notably, the postbiotic TSA 216 demonstrated inhibitory activity against *P. damselae* subsp. *piscicida*, *V. harveyi*, and *T. maritimum*. Previous studies have reported the antimicrobial activity of extracellular compounds from *Bacillus* species against these pathogens [51]. *P. damselae* subsp. *piscicida*, *V. harveyi*, and *T. maritimum* are responsible for causing photobacteriosis, vibriosis, and tenacibaculosis, respectively, in aquaculture. Broad-spectrum antibiotics and vaccines are currently used for their control [52, 53], but their efficacy varies depending on species and size [54].

Before being employed in human or animal feeding, postbiotics must undergo a safety evaluation, such as blood hemolytic activity [55]. In this sense, the assayed ECPs had γ-hemolytic, i.e., negative or no hemolytic activity. Our results are in line with several studies which reported lack of hemolytic activity in *Bacillus* species [56–58]. On the other hand, Bottone and Peluso [59] reported hemolytic activity of some *B. pumilus* strains.

Based on the hemolytic, hydrolytic, and antimicrobial activities of the postbiotics, we selected MICRO 169, TSA 216, CHL 216, and MICRO 216 for in vitro microalgae hydrolysis.

The in vitro hydrolysis of the four species of microalgae and the blend of them with the ECPs selected was carried out. The protein degradation coefficient (CPD) calculated from the electrophoresis gels evidenced that microalgal protein was easily hydrolyzed by the ECP proteases, as clear decrease in the optical density in the proteinograms together with a rapid increase in CPD occurs during the first minutes of in vitro assays. MICRO 169 and MICRO 216 ECPs produce the highest protein hydrolysis, which agree with the high endo-protease activity measured in these extracts.

The degree of proteolysis depends on the adequate protein bio-accessibility but also on the availability of suitable exo-proteases, capable of degrading larger polypeptides into free amino acids. The quantification of released amino acids allows estimating both the bio-availability of microalgal protein and the capacity of the ECP exo-proteases to release amino acids from the amino and carboxyl ends of polypeptide chains [8].

We found that ECP exo-proteases released around 50% of the amino acids of *T. galbana* and *A. platensis* protein after 120 min of in vitro hydrolysis. The differences observed in the amino acid released might be due to variations in the protein composition among microalgae species. The different protein conformation can allow or hinder the access of ECP peptidases, thus influencing the final amino acid bio-availability [8, 60]. MICRO 216 and MICRO 169 ECPs produced a higher release of amino acids, which is derived from the higher exo-peptidase enzyme activity in these two ECPs compared to TSA 216 and CHL 216 ECPs.

The ability of ECPs to hydrolyze the microalgae cell walls influences the accessibility of enzymes to intracellular compounds. In this sense, the quantification of reducing sugars released provides valuable information about the hydrolytic capacity of the ECP carbohydrases. The results obtained reflect a modest contribution of ECPs at this point, owing to the values of free sugars released that were almost negligible, which evidenced a scarce capacity of ECP carbohydrases to hydrolyze the microalgae cell walls.

Moving on to the physiological effects of these compounds when included in practical diets, the in vivo trial revealed that incorporating MICRO 169 along with microalgae into the aquafeeds of gilthead seabream juveniles, irrespective of the inclusion method or dosage, did not adversely affect growth performance and somatic indices, suggesting proper feed assimilation and metabolization without any imbalance at a general physiological level. The increase in intestinal length index associated with microalgae diets [61, 62] was particularly evident in the E10-M group by an indirect relationship between higher FE associated with the shortest intestines, thus demonstrating a clear and interesting improvement in gut functionality and intestinal well-being that would be very promising and necessary to elucidate in longer feeding periods. This will be more important in fish feed, as herein, with diets that cover the nutritional requirements and not only include a high proportion of plant raw materials as a current trend used in the industry but also when fish are maintained under sub-optimal conditions of low temperatures during autumn–winter in a short-/medium-term feeding trial. This issue has been previously described with other pre-, pro-, or postbiotics used in aquafeeds [63, 64], where this trend can potentially be correlated with the high proteolytic as well as gelatinase and caseinase activity observed in MICRO 169, which suggests enhanced protein degradation capabilities, leading to improved nutrient availability and absorption. In terms of metabolic response, the absence of significant differences in both plasma and liver, a priori, would indicate that the experimental diets did not impair important metabolic processes in the specimens of these groups. In fact, hepatic triglycerides of the MICROALGAE and E10-M groups show an interesting trend, with a lowering effect in the accumulation of lipids in the liver, reducing the risk of hepatic steatosis [65]. In groups E10-V and E5-V, these effects have not been observed, suggesting that this method of inclusion may not facilitate the assimilation and effects caused by microalgae. Another interesting result is circulating cortisol, as a parameter of welfare, which is not increased by including microalgae in the diet, contrary to what was previously described [62, 66], probably due to the addition of postbiotics. These interesting results demonstrate that the incorporation of ECPs in the diets did not stimulate the innate immune system under basal/normal conditions in the culture, thus avoiding an impairment that could be interpreted as a cause or consequence of metabolic orchestration and reorganization by higher energetic demands.

Finally, the evaluation of the activity of digestive enzymes is used to analyze the adaptations of cultivated species to variations in the composition of nutrients and type of ingredients used in artificial diets, since it represents a very reliable marker of digestive and intestinal absorption capacity, as well as an indicator of the nutritional status of fish [40, 67]. In this work, fish fed with diets supplemented with microalgae and ECP showed higher levels of the digestive enzyme activities studied, especially in the case of fish fed with the E10-M diet. The increase in the activity levels of certain digestive enzymes after the inclusion of microalgae biomass in diets has been described in several studies with different fish species [20, 68]. However, the increase in the activity of digestive enzymes observed in this work cannot be explained only by the addition of microalgae in feed, but part of the improvement in the digestive capacity of these fish is associated with the inclusion of ECP. However, due to the lack of information about the inclusion of extracellular products in aquaculture feeds, it is necessary to continue researching in this regard, with the aim of deepening and expanding knowledge in this aspect.

## Conclusion

In this study, we reported the significant influence of microalgae on the culture media for obtaining extracellular products from *B. pumilus* strains. Notably, these products exhibit diverse activities depending on the specific microalga or mix used for cultivation. Among the various conditions explored, *B. pumilus* UMA 169 grown on a medium supplemented with *M. gaditana* (MICRO 169) stood out, prompting its inclusion in the in vivo experiment with *S. aurata*. The results from the in vivo experiment demonstrated that, despite the absence of significant differences in terms of growth, nutrient utilization from the feed, or the metabolic response of the fish, the incorporation of MICRO 169 in the feeds led to a noteworthy improvement in digestive enzymatic activity. However, further studies are imperative to investigate the mechanisms underlying these results and to explore additional potential applications of ECPs from this strain as a potential postbiotic in aquaculture.

## Supplementary Information

Below is the link to the electronic supplementary material.

Supplementary file1 (DOCX 454 kb)

Supplementary file2 (DOCX 18 kb)

## Data Availability

Data will be made available on request.
