# Supplementary material for: Microalgal and Cyanobacterial Biomasses Modified the Activity of Extracellular Products from Bacillus pumilus: An In Vitro and In Vivo Assessment
Source: Probiotics Antimicrob Proteins. 2024 Sep 11;17(4):2179–96. doi: 10.1007/s12602-024-10350-z (PMC12405380; doi:10.1007/s12602-024-10350-z)
Supplement: Supplementary file 1 — Supplementary file1 (DOCX 454 kb) [file 12602_2024_10350_MOESM1_ESM.docx]

| 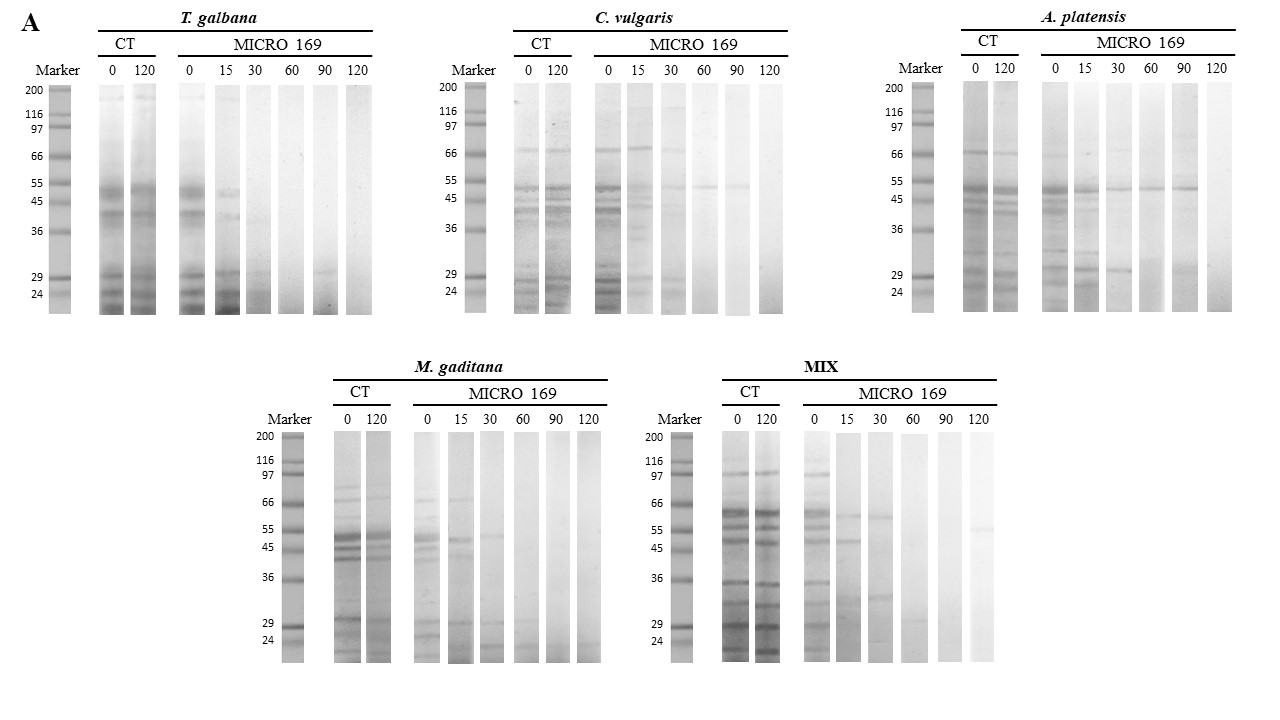 |
| --- |
| 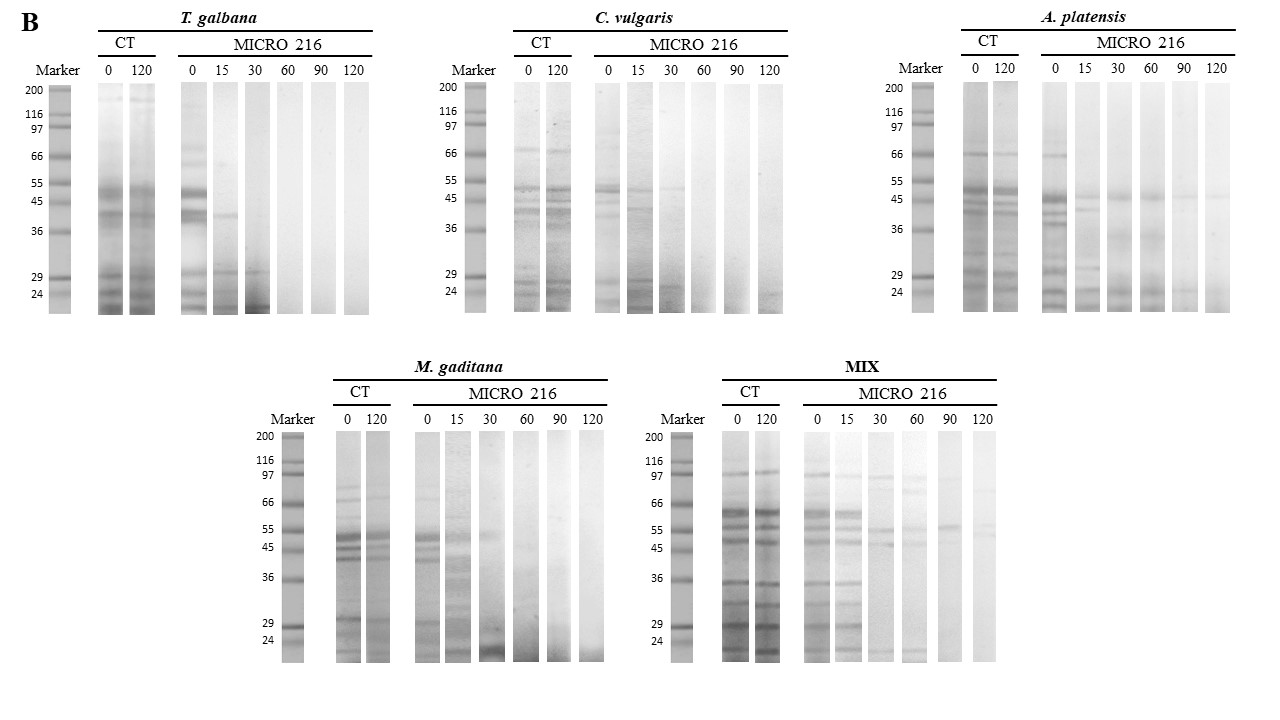 |
| 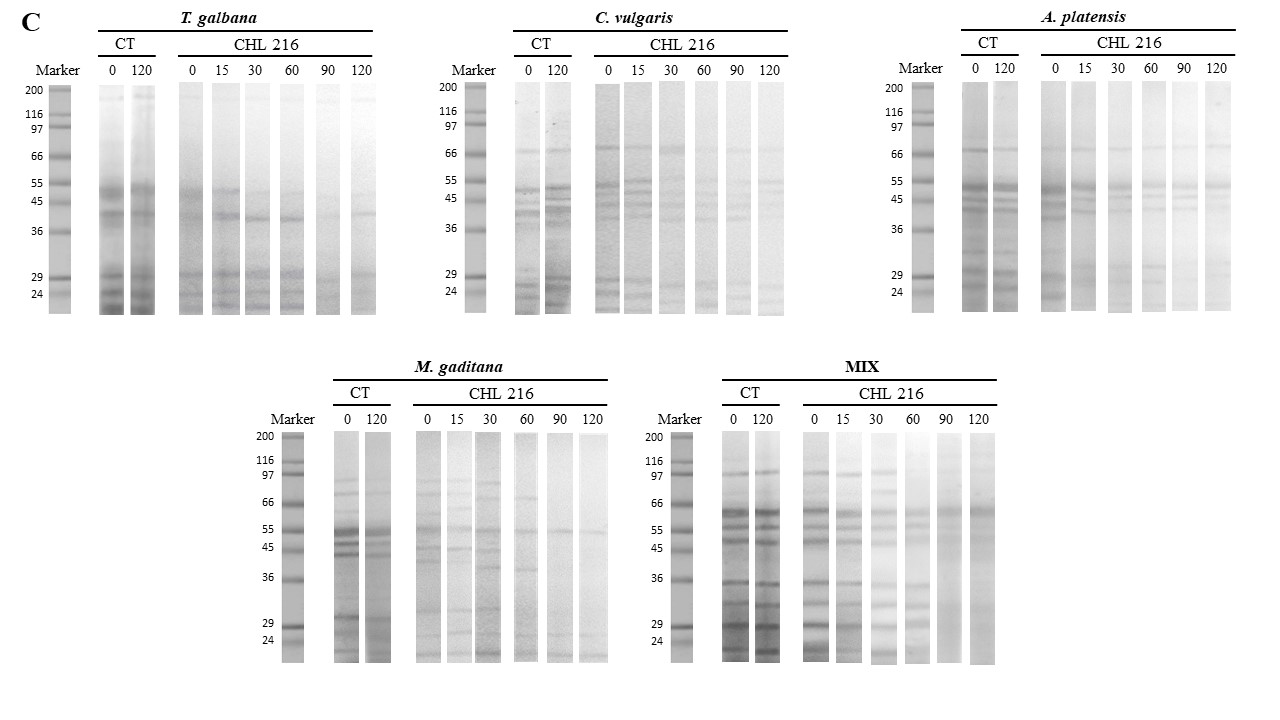 |
| 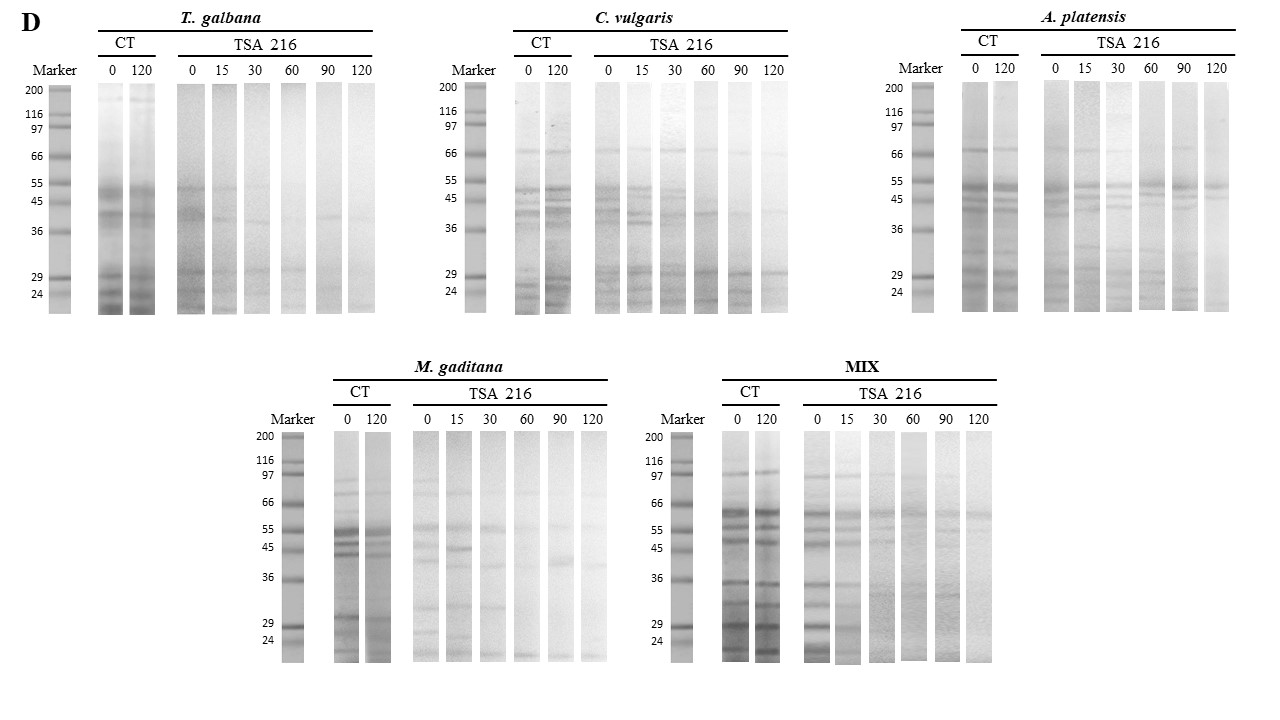 |

**Supplementary Figure 1.** Time-course of *in vitro* proteolysis of *T. galbana, C. vulgaris, A. platensis, M. gaditana*, and the mix of microalgae by the action of the different ECPs evaluated (A: MICRO 169; B: MICRO 216; C: CHL 216; D: TSA 216). CT corresponds to blank assays performed in the absence of ECPs. The molecular weights of the main protein fractions are indicated to the left of the marker and the initial CT time lane.
