# Supplementary material for: Microalgal and Cyanobacterial Biomasses Modified the Activity of Extracellular Products from Bacillus pumilus: An In Vitro and In Vivo Assessment
Source: Probiotics Antimicrob Proteins. 2024 Sep 11;17(4):2179–96. doi: 10.1007/s12602-024-10350-z (PMC12405380; doi:10.1007/s12602-024-10350-z)
Supplement: Supplementary file 2 — Supplementary file2 (DOCX 18 kb) [file 12602_2024_10350_MOESM2_ESM.docx]

**Supplementary Table 1**. Ingredient and proximate composition of the experimental diets.

|  | **CT** | **MICROALGAE** | **E-10M** | **E-5V** | **E-10V** |
| --- | --- | --- | --- | --- | --- |
| *Ingredients* (g kg^-1^ on dry basis) | | | | | |
| Fishmeal LT94 ^1^ | 10.0 | 10.0 | 10.0 | 10.0 | 10.0 |
| Soybean protein concentrate ^2^ | 15.0 | 14.0 | 14.0 | 14.0 | 14.0 |
| Wheat gluten ^3^ | 17.0 | 17.0 | 17.0 | 17.0 | 17.0 |
| Pea protein concentrate ^4^ | 5.0 | 5.0 | 5.0 | 5.0 | 5.0 |
| Soybean meal ^5^ | 20.0 | 18.5 | 18.5 | 18.5 | 18.5 |
| Wheat meal ^6^ | 14.14 | 12.54 | 12.54 | 12.54 | 12.54 |
| Fish oil ^7^ | 7.0 | 7.0 | 7.0 | 7.0 | 7.0 |
| Soybean oil ^8^ | 4.5 | 4.1 | 4.1 | 4.1 | 4.1 |
| Rapeseed oil ^9^ | 4.5 | 4.0 | 4.0 | 4.0 | 4.0 |
| Vitamin and mineral premix ^10^ | 1.0 | 1.0 | 1.0 | 1.0 | 1.0 |
| Vitamin C ^11^ | 0.05 | 0.05 | 0.05 | 0.05 | 0.05 |
| Vitamin E ^125^ | 0.01 | 0.01 | 0.01 | 0.01 | 0.01 |
| Methionine ^13^ | 0.50 | 0.5 | 0.5 | 0.5 | 0.5 |
| Monocalcium phosphate ^14^ | 1.30 | 1.30 | 1.30 | 1.30 | 1.30 |
| Microalgal biomass ^15^ | - | 5.0 | 5.0 | 5.0 | 5.0 |
| ECPs (mL kg^-1^) ^16^ | - | - | 10.0 | 5.0 | 10.0 |
| *Proximate composition* (%) | | | | | |
| Crude protein | 46.56 ± 0.10 | 46.66 ± 0.41 | 46.90 ± 0.17 | 46.61 ± 0.35 | 47.11 ± 0.27 |
| Total lipids | 19.86 ± 0.33 | 20.16 ± 0.27 | 19.89 ± 0.28 | 19.92 ± 0.24 | 19.88 ± 0.12 |
| Ash | 5.57 ± 0.04 | 6.00 ± 0.02 | 6.12 ± 0.05 | 6.00 ± 0.02 | 6.01 ± 0.04 |
| Moisture | 6.28 ± 0.09 | 6.51 ± 0.07 | 6.43 ± 0.07 | 6.14 ± 0.17 | 6.42 ± 0.25 |

Dietary codes: **CT**: diet control without ECP and microalgae. **MICROALGAE**: diet supplemented with 5 % of a blend of microalgae. **E-10M**: microalgae-supplemented diet enriched with the ECPs-nanoparticles (10 mL kg^-1^). **E-5V**: the microalgae-supplemented diet enriched with the ECPs solution (5 mL kg^-1^). **E-10V**: the microalgae-supplemented diet enriched with the ECPs solution (10 mL kg^-1^).

^1^(protein: 69.4 %; lipid: 12.3 %), Norsildemel (Bergen, Norway); ^2^ Soycomil, 60 % crude protein, 1.5 % crude lipid (ADM, Poland). ^3^ 78 % crude protein (Lorca Nutrición Animal SA, Murcia, Spain). ^4^ Pea protein concentrate, 85 % crude protein, 1.5 % crude lipid (Emilio Peña SA, Spain). ^5^ protein: 49.6 %, Lorca Nutrición Animal SA (Murcia, Spain). ^6^ Local provider (Almería, Spain). ^7^ AF117DHA (Afamsa, Spain). ^8, 9^ Soybean and rapeseed oils (Aceites el Niño, Spain). ^10^ *Lifebioencapsulation* SL (Almería, Spain). Vitamins (mg kg^-1^): vitamin A (retinyl acetate), 2,000,000 UI; vitamin D3 (DL-cholecalciferol), 200,000 UI; vitamin E (Lutavit E50), 10,000 mg; vitamin K3 (menadione sodium bisulphite), 2,500 mg; vitamin B1(thiamine hydrochloride), 3,000 mg; vitamin B2 (riboflavin), 3,000 mg; calcium pantothenate, 10,000 mg; nicotinic acid, 20,000 mg; vitamin B6 (pyridoxine hydrochloride), 2,000 mg; vitamin B9 (folic acid), 1,500 mg; vitamin B12 (cyanocobalamin), 10 mg vitamin H (biotin), 300 mg; inositol, 50,000 mg; betaine (Betafin S1), 50,000 mg. Minerals (mg kg-1): Co (cobalt carbonate), 65 mg; Cu (cupric sulphate), 900 mg; Fe (iron sulphate), 600 mg; I (potassium iodide), 50 mg; Mn (manganese oxide), 960 mg; Se (sodium selenite), 1 mg; Zn (zinc sulphate) 750 mg; Ca (calcium carbonate), 18.6 %; (186,000 mg); KCl, 2.41 %; (24,100 mg); NaCl, 4.0% (40,000 mg). ^11^ TECNOVIT, Spain. ^12, 13, 14^ Lorca Nutrición Animal SA (Murcia, Spain). ^15^ Blend of microalgae (25 % *C. vulgaris*, 25 % *A. platensis* and 50 % *M. gaditana*).
